# Supplementary material for: Determinants of Food Choice and Perceptions of Supermarket-Based Nudging Interventions among Adults with Low Socioeconomic Position: The SUPREME NUDGE Project
Source: Int J Environ Res Public Health. 2021 Jun 7;18(11):6175. doi: 10.3390/ijerph18116175 (PMC8201246; doi:10.3390/ijerph18116175)

Supplementary File 1. Topic list

| Topic | Interviewing questions |
| --- | --- |
| 1. Account of daily life | **GENERAL**<br>- How would you rate your life at this moment in time?<br>- Which factors in your life contribute to this? (e.g. physical/mental health, financial, work, social life)<br><br>**COMMUNITY**<br>- Can you describe your living situation?<br>- Do you have close relations with family / friends? What do they mean to you?<br>- How often do you meet up with them?<br>- When do you meet up with them?<br>- What do you do when you meet up with them? Why?<br><br>**ACTIVITIES [pick day before interview as guidance]**<br>*Daily schedule*<br>- What time did you get up? Why at that time?<br>- What did you do once you got out of bed?<br>- Did you have breakfast? Why (not)? What did you have for breakfast? With whom?<br>- At what time did you leave home?<br>- When leaving home, did you bring some food with you? Why (not)?<br>- Did you have lunch? At what time? What did you have? Where do you get your lunch?<br>- At what time did you get home?<br>- Did you have dinner? Why (not)? What did you have for dinner? With whom? Why?<br><br>*Food acquisition*<br>- Did you acquire any food that day?<br>- Where do you acquire food (e.g. supermarket, market)? Why there? Do you know the employees there?<br>- When do you normally acquire food? Weekly, daily?<br>- Do you enjoy acquiring food? Why (not)?<br>- How do you acquire food? Do you use of a grocery list? Do you consider promotions?<br>- Can you describe how a shopping trip looks like?<br><br>*Work*<br>- How did you get to work? Why do you choose that mode of transportation?<br>- Can you describe the type of work you do?<br>- Can you describe your working pattern? Is it constant or irregular?<br>- Do you enjoy your work? Why (not)?<br><br>*Leisure time*<br>- Do you have any spare time? When do you have spare time?<br>- What do you like to do in your spare time? With whom?<br>- Why do you enjoy that? |
| 2. Account of dietary behaviours | - How do you value a healthy diet? Is it important to you?<br>- What does a healthy diet mean to you?<br>- Do you consider yourself to have a healthy diet?<br>- Why do you eat what you eat?<br>➢ Health effects<br>➢ Taste<br>➢ Price<br>➢ Social network<br>➢ Habit<br>➢ Emotions<br>➢ Cultural influences<br>➢ Special occasions<br>➢ Stress |

| | |
|---|---|
| 3. Enablers and barriers | - What makes it easier for you to eat healthily? Why?<br>Probe, factors related to:<br>o Individual<br>o Lifestyle<br>o Community<br>o Local economy<br>o Activities<br>o Built environment<br>o Natural environment<br>- What makes it difficult for you to eat healthily? Why?<br>Probe, factors related to:<br>o Individual<br>o Lifestyle<br>o Community<br>o Local economy<br>o Activities<br>o Built environment<br>o Natural environment |
| 4. Needs for the promotion of healthy dietary behaviour | *The first part should really anticipate on previous answers of the interviewee. Anticipate on previously mentioned aspects of daily routine; factors that influence eating behaviours (e.g. price, taste, etc.); and barriers that make it difficult to eat healthily.*<br>o You previously indicated … What could help you to …?<br><br>*For the second part, we present the interviewee with graphical cards of possible interventions in the supermarket that promote a healthy lifestyle (e.g. including visualized nudges and pricing interventions).*<br>o Pick one card which appeals to you to the most. Can you describe which elements appeal to you? And why do they appeal to you?<br>o Pick one card which doesn't appeal to you at all. Can you describe which elements you don't like about it? What would be the conditions that would make it more appealing to you? |

**Supplementary File 2.** Visual examples nudges.

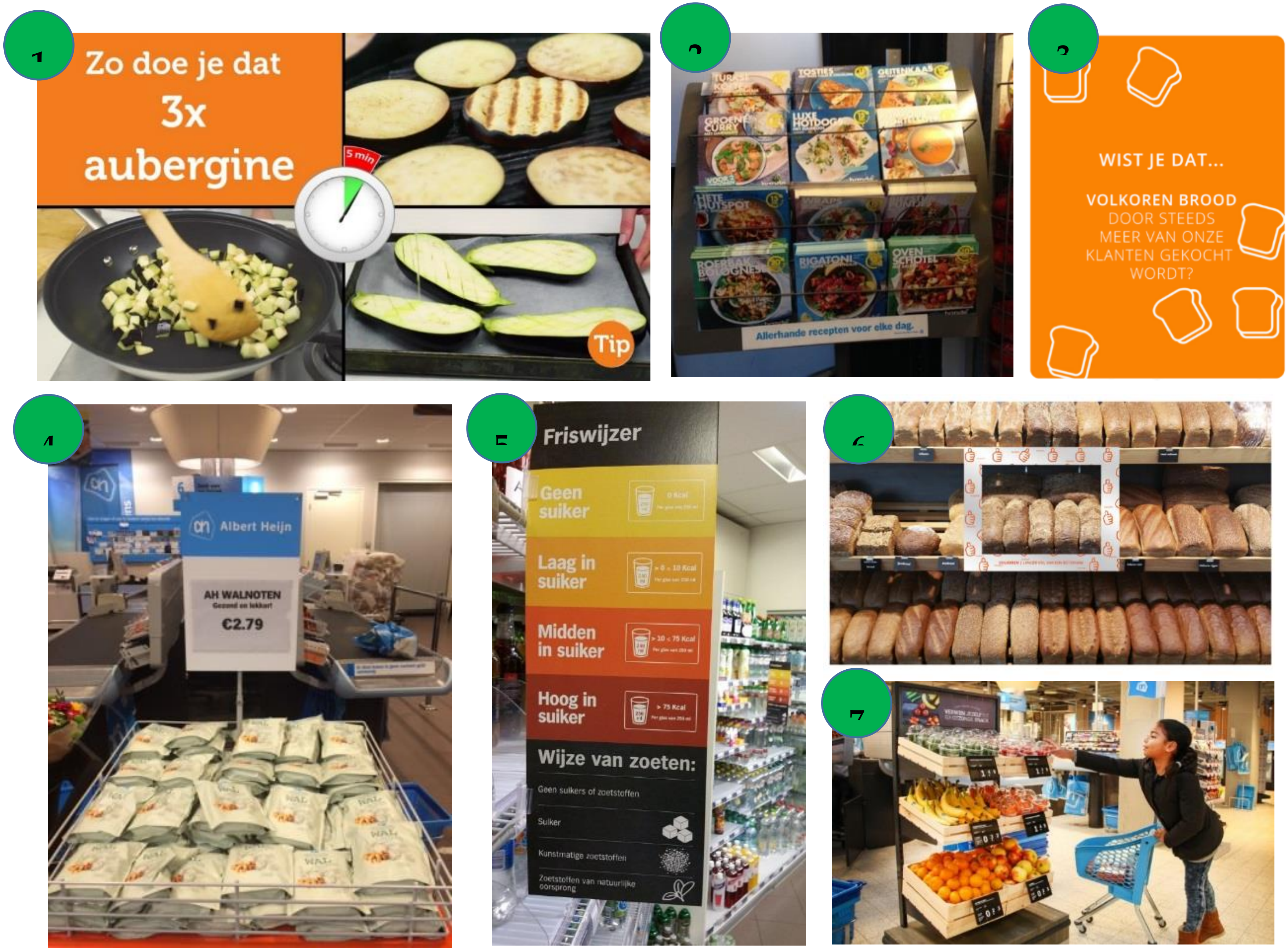

Supplement: Supplementary file 1 [file ijerph-18-06175-s001.zip › ijerph-1220243-supplementary.pdf]
